# Are sleep quality problems linked to pride in Tai Chi practitioners? Regulatory emotional self-efficacy and years of practice

**DOI:** 10.3389/fpsyg.2026.1933055

**Published:** 2026-10-06

**Authors:** Chengyan Sun, Qi Zhang, Lu Wang, Dan Li, Chi-Lun Tsai

**Affiliations:** 1 School of Wushu and Traditional Ethnic Sports, Jilin Sport University, Changchun, China; 2 College of Leisure and Social Sports, Jilin Sport University, Changchun, China; 3 Department of Sport Psychology, Leipzig University, Leipzig, Germany

**Keywords:** authentic pride, hubristic pride, moderated mediation, regulatory emotional self-efficacy, sleep quality problems, Tai Chi

## Abstract

**Background:**

Sleep quality is a central indicator of sleep health and is closely linked to emotional functioning. However, research has focused largely on negative affect, psychopathology, and impaired daytime functioning, leaving less known about whether sleep quality problems are associated with positive self-evaluative emotions. In the real-world context of Tai Chi practice, this study examined whether regulatory emotional self-efficacy (RESE) statistically accounted for the associations between sleep quality problems and authentic and hubristic pride among a predominantly young sample of Chinese Tai Chi practitioners, and whether years of Tai Chi practice moderated the association between sleep quality problems and RESE.

**Methods:**

A cross-sectional survey was conducted among 477 Chinese Tai Chi practitioners with regular and sustained practice experience, most of whom were young adults. Participants completed self-report measures, including the Pittsburgh Sleep Quality Index (PSQI), the RESE, and the Authentic and Hubristic Pride Scales (AHPS), and reported their years of Tai Chi practice experience. Mediation and moderated mediation analyses were conducted using PROCESS Models 4 and 7, controlling for gender and age.

**Results:**

Sleep quality problems were indirectly associated with lower authentic pride through lower RESE. The corresponding indirect association was not statistically significant for hubristic pride. Tai Chi experience moderated the first-stage association between sleep quality problems and RESE: the negative association was stronger among participants with longer Tai Chi experience, and the conditional indirect association with authentic pride was statistically significant at median and higher levels of experience.

**Conclusion:**

In this cross-sectional sample, lower perceived emotion-regulation capacity statistically accounted for part of the association between sleep quality problems and lower authentic pride. The corresponding indirect association was not statistically significant for hubristic pride. Tai Chi experience conditioned the association between sleep quality problems and RESE, but its direction was not consistent with a buffering interpretation.

## Introduction

Sleep quality is one of the core subjective markers of sleep health, with implications for how individuals recover, regulate affect, and function during the day. Existing research has primarily focused on negative affect, psychopathology, and cognitive consequences of poor sleep. Less is known about whether sleep quality problems are also associated with positive self-evaluative emotions—emotional states that involve how individuals appraise their own competence, agency, and capacity to manage everyday demands (Palmer et al., 2024; Parsons et al., 2022). Pride is a positive self-evaluative emotion rather than merely a pleasant affective state. It concerns what individuals take their actions, capacities, or personal condition to reveal about the self. Pride arises when individuals interpret an outcome, action, or personal state as reflecting something valuable about the self (Tangney et al., 2007; Tracy and Robins, 2004). It is associated with appraisals of agency, competence, self-worth, motivation, and social standing (Tracy et al., 2023; Williams and DeSteno, 2008). Tracy and Robins (2007) distinguished authentic pride, which is associated with effort, achievement, and self-mastery, from hubristic pride, which is associated with arrogance, superiority, and inflated self-evaluation. Rather than treating pride as a unitary positive emotion, the present study distinguishes authentic and hubristic pride as forms of self-evaluation that may show different associations with sleep-related difficulties.

Previous research has typically situated pride within achievement-related, personality-based, and social-status processes. For example, Carver et al. (2010) showed that authentic pride was more closely associated with self-control, whereas hubristic pride was more closely related to impulsivity and aggression. Similarly, Cheng et al. (2010) linked the two facets of pride to different status-related pathways, with authentic pride being more closely aligned with prestige and hubristic pride with dominance. These findings position authentic and hubristic pride within different configurations of self-regulation, self-evaluation, and social orientation, rather than as stronger or weaker versions of the same positive emotion (Cheng et al., 2010; Tracy et al., 2023). Yet achievement and social status are not the only contexts in which people form self-relevant appraisals. Everyday bodily recovery may also shape how individuals experience their functioning in daily life, particularly in relation to emotional and self-regulatory demands (Goldstein and Walker, 2014; Palmer et al., 2024). Sleep disruption has been linked to poorer self-control and to altered responses to positive stimuli, suggesting that sleep-related recovery may be relevant to the self-evaluative processes involved in positive emotion (Guarana et al., 2021; Pilcher et al., 2015). This possibility may be particularly relevant to authentic pride, which is grounded in appraisals of effort, competence, agency, and accomplishment (Tracy and Robins, 2004, 2007). Hubristic pride, by contrast, is more closely characterized by global superiority, arrogance, and defensive self-enhancement; its relation to everyday self-regulatory functioning may therefore be less direct (Tracy and Robins, 2007). Accordingly, examining sleep quality problems in relation to both facets of pride provides a way to extend pride research beyond its traditional achievement- and status-related contexts.

### Sleep quality problems and self-regulatory functioning

Sleep quality problems refer to subjective difficulties in sleep-related recovery, including poor perceived sleep quality, delayed sleep onset, insufficient or disrupted sleep, and daytime dysfunction (Buysse et al., 1989). In this sense, sleep quality is not merely a physiological background variable, but a daily condition through which individuals experience restoration, energy, and regulatory capacity. Poorer sleep quality has been associated not only with fatigue, attention problems, weaker cognitive control, and impaired daytime functioning, but also with emotional reactivity and difficulties in restoring psychological balance (Buysse et al., 1989; Goldstein and Walker, 2014; Palmer et al., 2024). Sleep quality is also related to broader dispositional and self-evaluative functioning. Duggan et al. (2014) found that lower conscientiousness and higher neuroticism were among the strongest personality correlates of poorer sleep, and Stephan et al. (2018) further showed longitudinal links between personality traits and sleep quality across four prospective samples. In addition, Lemola et al. (2013) found that insomnia symptoms and short sleep duration were associated with lower self-esteem. Taken together, this literature suggests that sleep quality problems are embedded in broader patterns of emotional stability, self-regulation, and self-evaluation, rather than being limited to isolated sleep complaints. Because pride depends on appraisals of competence, agency, and self-worth, sleep-related difficulties may be relevant to how individuals evaluate their own functioning.

These two facets of pride reflect different forms of self-evaluative emotional functioning. Authentic pride is more closely linked to effort, competence, and self-mastery, whereas hubristic pride is more closely linked to superiority-oriented and defensive self-evaluation (Tracy and Robins, 2004, 2007). This distinction raises the possibility that sleep-related difficulties may not be associated with the two facets in the same way. When sleep-related recovery is insufficient, individuals may feel more fatigued, emotionally unstable, or less capable of managing internal demands effectively (Goldstein and Walker, 2014; Guarana et al., 2021; Palmer et al., 2024). Such experiences may be particularly relevant to self-evaluative emotional functioning, including how individuals appraise their own competence, agency, and self-mastery. For this reason, authentic and hubristic pride were examined as separate outcomes, while avoiding the assumption that differences in statistical significance would by themselves demonstrate distinct psychological pathways. Accordingly, the present study proposed the following hypotheses:

*H1a*. Sleep quality problems will be associated with authentic pride.

*H1b*. Sleep quality problems will be associated with hubristic pride.

### Regulatory emotional self-efficacy as a potential sleep–emotion pathway

Regulatory emotional self-efficacy (RESE) refers to individuals’ perceived capability to manage, express, and recover from emotional states (Bandura, 1977; Caprara et al., 2008). It is not a measure of the specific strategies individuals use when regulating emotion, such as reappraisal, suppression, attentional deployment, or acceptance (Gross, 1998, 2015; Lincoln et al., 2022). Nor does it describe a broad and fixed personality disposition. Rather, RESE concerns whether individuals believe that they can remain effective when they encounter emotional discomfort, stress, or fluctuation.

Research on sleep and emotion provides a basis for considering RESE within this framework. A narrative review of insomnia and sleep-quality research has likewise identified emotion regulation and affect dynamics as central processes in the association between disturbed sleep and daytime emotional functioning (Meneo et al., 2023). Poor sleep and sleep loss have been associated with heightened emotional reactivity, altered emotion experience, and less effective emotion-regulation functioning (Goldstein and Walker, 2014; Palmer et al., 2024; Parsons et al., 2022). Emotion regulation has also been identified as a statistical mediator between sleep quality and stress- and aggression-related outcomes (Demichelis et al., 2023). A recent meta-analysis further found that insomnia was robustly associated with emotion dysregulation across both regulatory strategies and broader dispositional difficulties (Samea et al., 2025). These findings do not establish that sleep quality problems reduce regulatory emotional self-efficacy. They do, however, locate sleep-related recovery within the conditions under which individuals manage, interpret, and evaluate their emotional functioning. A previous study among Chinese college students reported that regulatory emotional self-efficacy and dormitory interpersonal relationships sequentially mediated the association between sleep quality and social anxiety (Sun, 2023). However, whether RESE is also involved in the association between sleep quality problems and self-conscious emotions such as pride remains unclear.

As a self-evaluative emotion, pride may also be related to perceived capability in managing emotional states (Tracy and Robins, 2007). RESE is not treated here as a generic mediator that automatically explains the effects of poor sleep. It is examined as an appraisal-level construct that may statistically account for associations between subjective sleep-related recovery and two forms of pride. Authentic and hubristic pride were analysed separately, without assuming that an observed difference in significance would demonstrate a statistically established difference between the two pathways. Based on this reasoning, the present study proposed the following hypotheses:

*H2*. Sleep quality problems will be associated with RESE.

*H3a*. RESE will be associated with authentic pride.

*H3b*. RESE will be associated with hubristic pride.

*H4a*. Sleep quality problems will be indirectly associated with authentic pride through RESE.

*H4b*. Sleep quality problems will be indirectly associated with hubristic pride through RESE.

### Tai Chi experience as a contextual condition

The proposed indirect pathway may also vary according to Tai Chi practice experience, which was examined as a potential contextual condition. Tai Chi is a mind–body practice that combines slow coordinated movement, breathing regulation, attentional control, postural balance, and awareness of bodily states (Wang et al., 2010; Wayne and Kaptchuk, 2008; Yeh et al., 2014). Research has linked Tai Chi practice to several domains relevant to the present model, including sleep quality (Khaleghi and Ahmadi, 2024; Si et al., 2020; Siu et al., 2021; Siu et al., 2025), psychological wellbeing, stress reduction, working memory, and emotion regulation (Wang et al., 2010; Wang et al., 2023). This body of work makes Tai Chi a relevant context for examining how bodily recovery and emotional self-regulation may become jointly salient in everyday functioning. Longer Tai Chi experience may reflect more sustained exposure to practices involving breathing, movement, balance, and attention to internal bodily states; these features are conceptually related to interoceptive and body-awareness processes that may be relevant to emotional functioning (Kraemer et al., 2021; Mehling et al., 2012; Yeh et al., 2014). Together, these considerations provide a basis for examining whether accumulated Tai Chi experience conditions the proposed sleep quality problems–RESE pathway and, consequently, the indirect associations with authentic and hubristic pride. The following hypotheses were proposed:

*H5a*. Tai Chi experience will moderate the indirect association between sleep quality problems and authentic pride through RESE.

*H5b*. Tai Chi experience will moderate the indirect association between sleep quality problems and hubristic pride through RESE.

### The current study

The present study examined associations between sleep quality problems, RESE, and two facets of pride among Tai Chi practitioners. At a conceptual level, the model brings together three related subjective dimensions of functioning: perceived difficulties with sleep-related recovery, perceived capability to manage emotional states, and self-evaluative emotion. RESE was examined as a potential psychological correlate of the association between sleep quality problems and self-evaluative emotion. Authentic and hubristic pride were modelled as separate outcomes. Years of Tai Chi practice were examined as a non-directional moderator of the association between sleep quality problems and RESE. Practice years were interpreted as a contextual indicator of accumulated exposure rather than as a direct measure of mindfulness, interoceptive awareness, or practice quality. The hypothesized models are presented in Figure 1.

**Figure 1:**
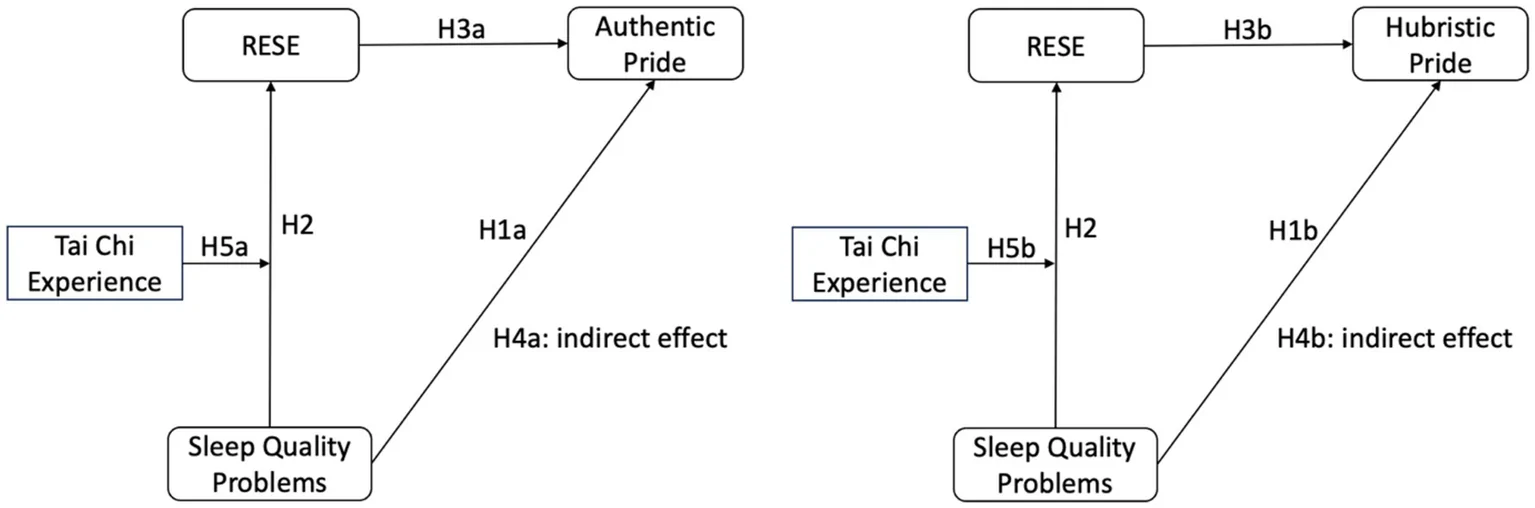
Hypothesized moderated mediation models. RESE = regulatory emotional self-efficacy.

## Methods

### Participants

Participants were Tai Chi practitioners recruited in China in January 2026 through site-based recruitment from multiple Tai Chi clubs and class-based Tai Chi courses, including university and community programs. Eligibility criteria were: (a) age of 18 years or older; (b) current engagement in Tai Chi practice; (c) at least 1 year of Tai Chi practice experience; (d) regular Tai Chi practice, operationally defined as practicing at least three times per week; (e) a typical practice duration of at least 40 min per session; and (f) no prolonged interruption in Tai Chi practice during the previous 6 months. The frequency and duration criteria were informed by prior Tai Chi exercise-prescription research and were used to ensure that participants had regular and sustained engagement with Tai Chi, rather than only brief, occasional, or discontinued exposure (Huang et al., 2021).

The final sample comprised 477 participants, including 350 men (73.4%) and 127 women (26.6%). Participants’ mean age was 21.19 years (SD = 4.08; range = 18–49). The age distribution was strongly concentrated among younger adults, with 83.2% of participants aged 18–22 years and only three participants aged 40 years or older. Tai Chi experience was operationalized as years of Tai Chi practice. Participants reported an average of 6.64 years of Tai Chi practice experience (SD = 3.27; range = 1–15). Overall, the sample was predominantly young and consisted of Chinese Tai Chi practitioners with regular and sustained practice experience in organized club- and class-based settings.

### Measurements

#### Sleep quality problems

Sleep quality problems were assessed using the Pittsburgh Sleep Quality Index (PSQI; Buysse et al., 1989). Specifically, we employed the Chinese version of the PSQI (Liu et al., 1996), which is widely used to assess subjective sleep quality and sleep-related problems. The PSQI captures multiple aspects of sleep over the previous month, including subjective sleep quality, sleep latency, sleep duration, habitual sleep efficiency, sleep disturbances, use of sleep medication, and daytime dysfunction. In the present study, the PSQI global score was treated as an index of poor sleep quality and sleep-related problems. The seven component scores were summed to compute a global PSQI score ranging from 0 to 21, with higher scores indicating poorer sleep quality and greater sleep-related problems. Internal consistency was acceptable for this multidimensional index (Cronbach’s *α* = 0.686).

#### Regulatory emotional self-efficacy

Regulatory emotional self-efficacy was assessed using the Regulatory Emotional Self-Efficacy Scale (RESE; Caprara et al., 2008). Specifically, we employed the 12-item Chinese version of the RESE (Zhang et al., 2010), which comprises three dimensions: perceived self-efficacy in expressing positive emotions, managing sadness or distress, and controlling anger. The RESE conceptualizes RESE as individuals’ perceived capability to effectively regulate and express emotional responses across different affective situations. In the present study, RESE was treated as a perceived psychological self-regulatory resource associated with adaptive functioning by helping individuals maintain emotional balance and manage negative emotional experiences. Participants responded on a 5-point Likert scale, with higher scores indicating greater perceived RESE. We computed an overall RESE score by averaging all 12 items. Internal consistency was excellent (Cronbach’s *α* = 0.962).

#### Pride

Pride was assessed using the Authentic and Hubristic Pride Scales (AHPS; Tracy and Robins, 2007). Specifically, we employed the Chinese version of the AHPS (Yang and Wang, 2011), which consists of 14 items and measures two theoretically distinct facets of pride: Authentic Pride and Hubristic Pride, with seven items for each subscale. Authentic pride reflects achievement-based, effort-related, and self-confident feelings, whereas hubristic pride reflects arrogance, self-aggrandizement, and superiority-oriented self-evaluations. In the present study, the AHPS was used to assess dispositional pride-proneness, that is, participants’ tendency to endorse authentic- and hubristic-pride experiences, rather than momentary pride states. Participants responded on a 5-point scale, with higher scores indicating stronger endorsement of the corresponding pride-proneness tendency. Separate scores were computed for authentic pride and hubristic pride by averaging the items of each subscale. Internal consistency was excellent for both authentic pride (Cronbach’s *α* = 0.973) and hubristic pride (Cronbach’s *α* = 0.987).

### Procedures and statistical analysis

Ethical approval for the study was obtained from the Institutional Ethics Committee of Jilin Sport University (Approval No. 2026008). Questionnaires were administered in online and offline formats and were completed anonymously. Prior to participation, participants were informed about the purpose of the study, the voluntary nature of participation, their right to withdraw at any time without penalty, and the confidential use of their responses for research purposes only. Informed consent was obtained from all participants before survey completion. No directly identifying information was collected.

All analyses were conducted using IBM SPSS Statistics (version 29.1; IBM Corp., Armonk, NY, USA) and the PROCESS macro for SPSS (version 4.2; Hayes, 2022). Descriptive statistics and Pearson correlations were computed for all study variables. Because all core constructs were assessed using self-report questionnaires at a single time point, Harman’s single-factor analysis was conducted as a diagnostic check of whether a single factor dominated the covariance structure. The seven PSQI component scores, 12 RESE items, and 14 AHPS items were entered into an unrotated one-factor principal component analysis. Mediation analyses were first performed using PROCESS Model 4 to test whether RESE mediated the associations of sleep quality problems with authentic and hubristic pride. Sleep quality problems were entered as the predictor in separate models, with authentic pride and hubristic pride examined as separate outcomes. Gender and age were included as covariates in the mediation models.

Moderated mediation analyses were then conducted using PROCESS Model 7 to examine whether Tai Chi experience moderated the first-stage paths from sleep quality problems to RESE. In these models, Tai Chi experience was specified as the moderator, and gender and age were included as covariates. Indirect and conditional indirect effects were estimated using 5,000 bootstrap resamples and 95% confidence intervals. Effects were considered significant when the confidence interval did not include zero. In the moderated mediation analyses, conditional effects were probed at the 16th, 50th, and 84th percentiles of Tai Chi experience, corresponding to low, moderate, and high levels of experience. The index of moderated mediation was used to determine whether the indirect association varied across levels of Tai Chi experience. Unstandardized coefficients are reported throughout. As a sensitivity analysis for multiple testing, the Benjamini–Hochberg FDR procedure (*q* = 0.05) was applied to eight focal conventional regression-path *p* values: sleep quality problems → RESE; the total and direct associations of sleep quality problems with authentic pride; RESE → authentic pride; the total and direct associations of sleep quality problems with hubristic pride; RESE → hubristic pride; and the Sleep Quality Problems × Tai Chi experience interaction → RESE. Covariate effects, conditional simple slopes, bootstrap-based indirect effects, and indices of moderated mediation were not included in the FDR family; the latter two were evaluated using their 95% bootstrap confidence intervals.

## Results

### Descriptive statistics and correlation analysis

Descriptive statistics and bivariate correlations are presented in Table 1. Sleep quality problems were negatively associated with RESE (*r* = −0.225) and authentic pride (*r* = −0.244), but were not significantly associated with hubristic pride (*r* = 0.073). RESE showed a strong positive association with authentic pride (*r* = 0.641), but was not significantly associated with hubristic pride (*r* = 0.059).

**Table 1:** Descriptive analysis and correlations.

Variable	*M* (SD)	1	2	3	4
1. Sleep quality problems	3.59 (2.93)	1			
2. RESE	4.01 (0.86)	−0.225^**^	1		
3. Authentic pride	3.65 (0.96)	−0.244^**^	0.641^**^	1	
4. Hubristic pride	2.17 (1.23)	0.073	0.059	0.188^**^	1

RESE = regulatory emotional self-efficacy. ^*^p < 0.05, ^**^p < 0.01.

### Assessment of common method variance

Harman’s single-factor analysis was conducted as a diagnostic assessment of whether a single factor dominated the covariance structure. The Kaiser–Meyer–Olkin value was 0.931, and Bartlett’s test of sphericity was significant, *χ*^2^(528) = 21,924.44, *p* < 0.001. The first unrotated factor accounted for 37.969% of the total variance, indicating that no single factor dominated the covariance structure.

### Testing for the mediation models

The mediating role of RESE was examined using PROCESS Model 4. Sleep quality problems were entered as the predictor, RESE as the mediator, and authentic pride and hubristic pride as outcomes in separate models. Gender and age were included as covariates in the mediation models.

As shown in Table 2, sleep quality problems were negatively associated with RESE, *b* = −0.063, SE = 0.014, *p* < 0.001, 95% CI [−0.090, −0.037]. For authentic pride, sleep quality problems remained directly associated with authentic pride after RESE was included, *b* = −0.035, SE = 0.012, *p* = 0.004, 95% CI [−0.059, −0.012]. RESE was positively associated with authentic pride, *b* = 0.691, SE = 0.040, *p* < 0.001, 95% CI [0.612, 0.770]. The estimated indirect association involving RESE was statistically significant, *b* = −0.044, BootSE = 0.010, 95% CI [−0.063, −0.024].

**Table 2:** PROCESS Model 4 mediation effects of regulatory emotional self-efficacy.

Regression equation		Fit index				Significance of regression coefficient			
Outcome	Predictor	*R*	*R* ^2^	*F*	*b*	SE	LLCI	ULCI	*t*
Common mediator model predicting RESE									
RESE	SQP	0.230	0.053	8.771	−0.063	0.014	−0.090	−0.037	−4.701^***^
	Gender				−0.021	0.081	−0.180	0.137	−0.266
	Age				0.009	0.010	−0.010	0.028	0.959
Total effect model									
AP	SQP	0.248	0.062	10.334	−0.079	0.015	−0.109	−0.050	−5.276^***^
	Gender				−0.054	0.090	−0.123	0.230	0.595
	Age				0.008	0.011	−0.013	0.029	0.722
Mediation model									
AP	SQP	0.650	0.422	86.253	−0.035	0.012	−0.059	−0.012	−2.936^**^
	RESE				0.691	0.040	0.612	0.770	17.169^***^
	Gender				0.068	0.071	−0.070	0.207	0.968
	Age				0.001	0.008	−0.015	0.018	0.163
Total effect model									
HP	SQP	0.137	0.019	3.028	0.040	0.020	0.001	0.078	2.030^*^
	Gender				−0.276	0.117	−0.506	−0.047	−2.363^*^
	Age				0.016	0.014	−0.012	0.043	1.126
Mediation model									
HP	SQP	0.156	0.024	2.941	0.047	0.020	0.007	0.086	2.331^*^
	RESE				0.108	0.067	−0.023	0.239	1.627
	Gender				−0.274	0.117	−0.503	−0.045	−2.347^*^
	Age				0.015	0.014	−0.013	0.042	1.056
Indirect effect					Effect	SE	LLCI	ULCI	
SQP → RESE → AP					−0.044	0.010	−0.063	−0.024	
SQP → RESE → HP					−0.007	0.005	−0.017	0.002	

AP = authentic pride; HP = hubristic pride; SQP = sleep quality problems; RESE = regulatory emotional self-efficacy; LLCI = lower limit of the 95% confidence interval; ULCI = upper limit of the 95% confidence interval. Mediation models were adjusted for gender and age. Indirect-effect estimates are based on 5,000 bootstrap resamples. Unstandardized coefficients are reported. ^*^p < 0.05, ^**^p < 0.01, ^***^p < 0.001.

For hubristic pride, sleep quality problems showed a small positive direct association with hubristic pride after RESE was included, *b* = 0.047, SE = 0.020, *p* = 0.020, 95% CI [0.007, 0.086]. RESE was not significantly associated with hubristic pride, *b* = 0.108, SE = 0.067, *p* = 0.104, 95% CI [−0.023, 0.239]. The corresponding indirect association involving RESE was not statistically significant, *b* = −0.007, BootSE = 0.005, 95% CI [−0.017, 0.002].

### Testing for the moderated mediation models

PROCESS Model 7 was used to examine whether Tai Chi experience moderated the first-stage association between sleep quality problems and RESE. Gender and age were included as covariates.

As shown in Table 3 and Figure 2, the interaction between sleep quality problems and Tai Chi experience significantly predicted RESE, *b* = −0.011, SE = 0.004, *t* = −2.559, *p* = 0.011, 95% CI [−0.019, −0.003]. Conditional effects showed that the negative association between sleep quality problems and RESE became stronger at higher levels of Tai Chi experience. Specifically, this association was not significant at relatively lower Tai Chi experience, *b* = −0.034, *p* = 0.063, but was significant at the median level, *b* = −0.077, *p* = 0.001, and at relatively higher Tai Chi experience, *b* = −0.109, *p* < 0.001 (see Figure 3).

**Table 3:** Moderated mediation models.

Model/effect	*b*	SE	*t*	*p*	LLCI	ULCI
Model for predicting RESE						
Gender	−0.039	0.087	−0.448	0.655	−0.211	0.133
Age	0.007	0.016	0.442	0.659	−0.024	0.038
Sleep quality problems	−0.002	0.024	−0.098	0.922	−0.050	0.045
Tai Chi experience	0.056	0.020	2.877	0.004	0.018	0.095
Sleep quality problems × Tai Chi experience	−0.011	0.004	−2.559	0.011	−0.019	−0.003
Conditional indirect effects for Sleep quality problems → RESE → AP						
Relatively lower experience (16th percentile = 3 years)	−0.024	0.016			−0.055	0.008
Median experience (50th percentile = 7 years)	−0.053	0.017			−0.088	−0.021
Relatively higher experience (84th percentile = 10 years)	−0.075	0.023			−0.122	−0.033
Index of moderated mediation	−0.007	0.003			−0.014	−0.001
Conditional indirect effects for Sleep quality problems → RESE → HP						
Relatively lower experience (16^th^ percentile = 3 years)	−0.004	0.004			−0.012	0.002
Median experience (50^th^ percentile = 7 years)	−0.008	0.006			−0.022	0.003
Relatively higher experience (84^th^ percentile = 10 years)	−0.011	0.009			−0.032	0.004
Index of moderated mediation	−0.001	0.001			−0.003	0.0003

RESE = regulatory emotional self-efficacy; AP = authentic pride; HP = hubristic pride. Conditional indirect effects were probed at the 16th, 50th, and 84th percentiles of Tai Chi experience. These values represent relatively lower, median, and relatively higher practice years within the present sample, rather than categorical groups. Unstandardized coefficients are reported.

**Figure 2:**
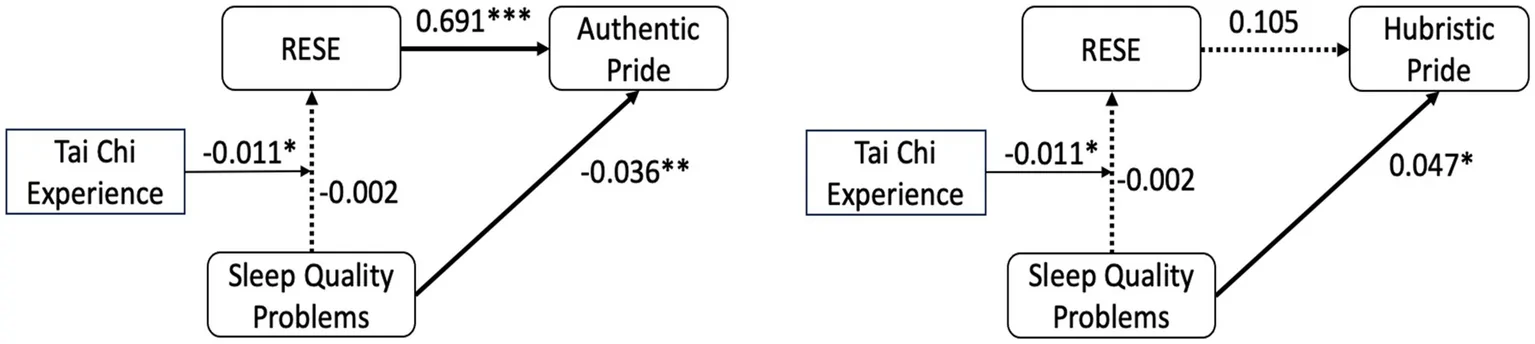
Results of the PROCESS Model 7 moderated mediation models. RESE = regulatory emotional self-efficacy. Unstandardized coefficients are shown. Solid lines indicate statistically significant paths, whereas dotted lines indicate nonsignificant paths. Gender and age were included as covariates but are not shown for visual clarity. ^*^p < 0.05. ^**^p < 0.01. ^***^p < 0.001.

**Figure 3:**
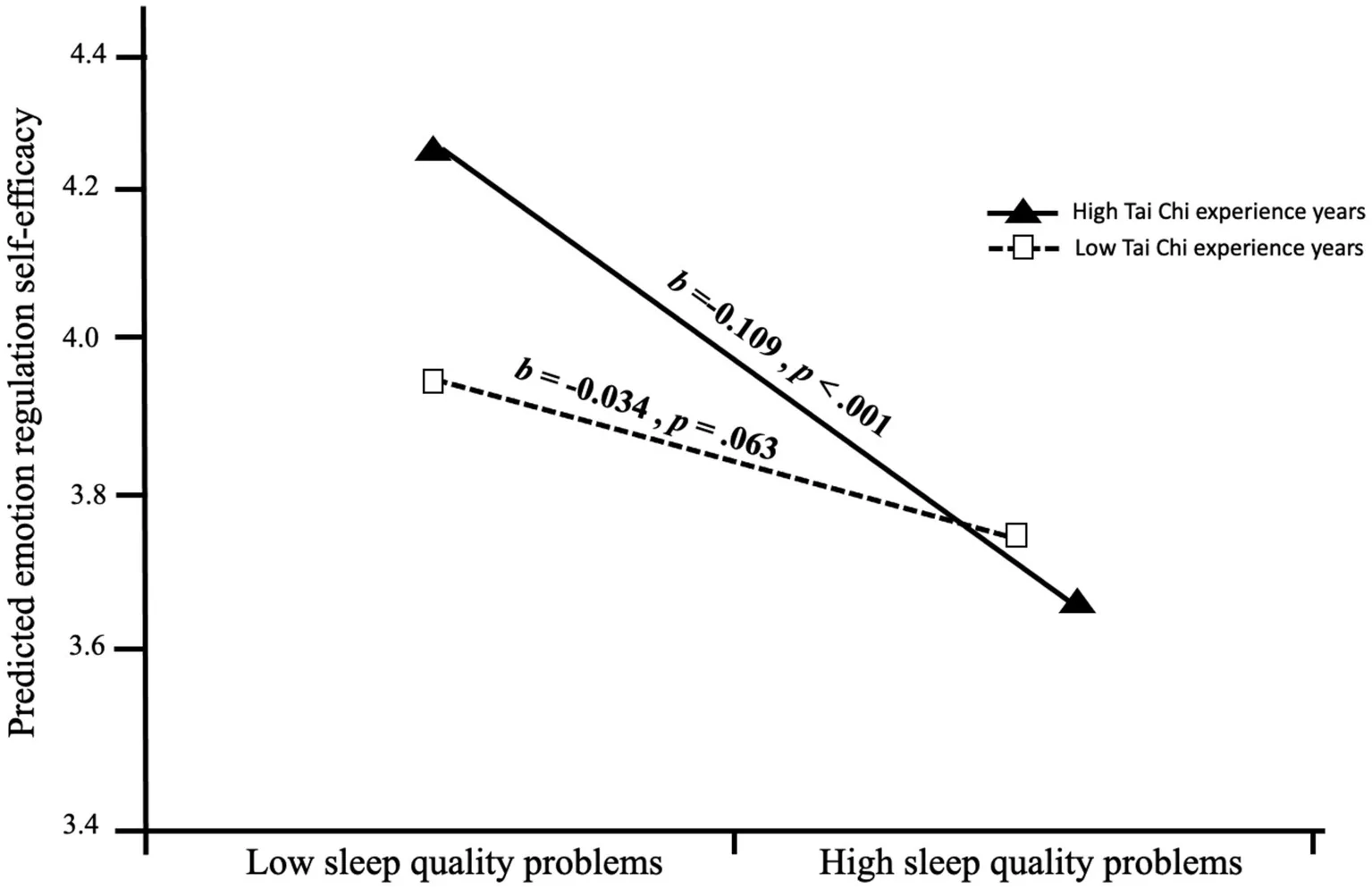
Conditional association between sleep quality problems and regulatory emotional self-efficacy at lower and higher levels of Tai Chi experience.

The moderated mediation results showed that the conditional indirect association between sleep quality problems and authentic pride through RESE was significant at the median and relatively higher levels of Tai Chi experience, but not at the relatively lower level. The index of moderated mediation was significant, *b* = −0.007, SE = 0.003, 95% CI [−0.014, −0.001], indicating that the indirect association differed across levels of Tai Chi experience. By contrast, the corresponding index of moderated mediation for hubristic pride was not significant, *b* = −0.001, SE = 0.001, 95% CI [−0.003, 0.0003]. Thus, the moderated indirect association through regulatory emotional self-efficacy was statistically significant for authentic pride, but not for hubristic pride.

The FDR sensitivity analysis did not alter the substantive interpretation of the focal regression-path findings. After Benjamini–Hochberg adjustment, the association between sleep quality problems and RESE remained statistically significant (FDR-adjusted *p* < 0.001). For authentic pride, both the total association with sleep quality problems (FDR-adjusted *p* < 0.001) and the direct association after RESE was included (FDR-adjusted *p* = 0.007) remained significant, as did the association between RESE and authentic pride (FDR-adjusted *p* < 0.001). The Sleep Quality Problems × Tai Chi experience interaction predicting RESE also remained significant (FDR-adjusted *p* = 0.017). For hubristic pride, both the total association with sleep quality problems (FDR-adjusted *p* = 0.049) and the direct association after RESE was included (FDR-adjusted *p* = 0.027) remained statistically significant, whereas the association between RESE and hubristic pride remained nonsignificant (FDR-adjusted *p* = 0.104). Bootstrap-based indirect associations and indices of moderated mediation were not included in the FDR procedure.

## Discussion

The present study examined whether sleep quality problems were associated with two forms of pride through RESE, and whether Tai Chi experience conditioned this statistical association. Sleep quality problems were indirectly associated with lower authentic pride through lower RESE, whereas the corresponding indirect association was not statistically significant for hubristic pride. Tai Chi experience also moderated the association between sleep quality problems and RESE. Rather than showing a protective pattern, the negative association between sleep quality problems and RESE was stronger among participants with more years of Tai Chi experience. Taken together, these findings position sleep-related recovery and perceived emotion-regulation capacity as correlates of self-evaluative emotional functioning in a real-world Tai Chi practice context.

First, the negative association between sleep quality problems and authentic pride supported H1a and is consistent with the broader literature on sleep and emotion regulation. Previous research has generally shown that poorer sleep quality is associated with heightened emotional reactivity, impaired psychological recovery, weaker self-regulatory capacity, and lower emotional stability (Goldstein and Walker, 2014; Palmer et al., 2024; Parsons et al., 2022; Tomaso et al., 2021). The present findings add to this line of work by contributing to the domain of self-conscious emotions by showing that sleep quality problems may be related not only to general emotional states, but also to how individuals evaluate their own competence, stability, and sense of self-control. Authentic pride is not merely a pleasant affective state. Rather, it is a positive self-evaluative emotion grounded in effort, competence, and self-mastery (Tracy and Robins, 2007). Individuals who reported more sleep quality problems may also have perceived themselves as less stable, effective, and in control, which may help explain why sleep quality problems were associated with lower authentic pride. From this perspective, the present findings extend prior work linking sleep quality to emotional, dispositional, and self-evaluative functioning by showing that sleep-related difficulties may also be relevant to self-conscious emotional experience, particularly authentic pride.

In contrast, sleep quality problems were not significantly associated with hubristic pride at the bivariate level, although a small positive association emerged after gender and age were controlled and remained significant after RESE was included in the mediation model. Thus, H1b received limited support in the covariate-adjusted models. This mixed pattern is consistent with, but does not by itself establish, the view that authentic and hubristic pride may have different psychological correlates. Authentic pride is more closely tied to effort, competence, and self-mastery, whereas hubristic pride is more often associated with superiority, inflated self-evaluation, defensive self-esteem, and status-oriented self-presentation (Cheng et al., 2010; Tracy and Robins, 2007). This pattern suggests that the association between sleep quality problems and hubristic pride may not be well explained by perceived emotion-regulation capacity. Whereas authentic pride appears to be more closely linked to self-regulatory appraisals such as perceived emotional competence and self-mastery, the small positive association observed for hubristic pride may reflect a different form of self-evaluation, one that is less dependent on regulatory efficacy and more closely tied to superiority-oriented or defensive self-appraisal. However, because the present analyses did not formally compare the strength of the paths across the two pride outcomes, this interpretation should remain cautious.

Furthermore, sleep quality problems were associated with lower RESE, supporting H2. This pattern can be understood from a resource-oriented perspective on self-regulation. Participants reporting more sleep quality problems may perceive themselves as having fewer psychological resources to maintain emotional stability, restore psychological balance, and manage internal states (Goldstein and Walker, 2014; Palmer et al., 2024). Importantly, RESE does not refer to the specific emotion-regulation strategies individuals use, but to their perceived capability to manage, alleviate, and recover from emotional states (Bandura, 1977; Caprara et al., 2008; Gross, 1998). RESE was positively associated with authentic pride, supporting H3a, whereas its association with hubristic pride was not significant; therefore, H3b was not supported. This pattern is conceptually compatible with the distinction between authentic and hubristic pride, although the present analyses did not formally test whether the associations with RESE differed across the two facets. RESE concerns perceived capability to manage and recover from emotional states (Bandura, 1977; Caprara et al., 2008), whereas authentic pride is organized around competence and self-mastery and hubristic pride around superiority-oriented and defensive self-evaluations (Cheng et al., 2010; Tracy and Robins, 2007). This conceptual contrast offers a plausible interpretation of the observed pattern, but it should be tested directly in future research.

With respect to mediation, sleep quality problems were indirectly associated with lower authentic pride through lower RESE, supporting H4a. This finding is consistent with the idea that sleep-related difficulties may be linked not only to poorer emotional states, but also to weaker self-efficacy beliefs regarding individuals’ ability to regulate emotions (Goldstein and Walker, 2014; Palmer et al., 2024; Parsons et al., 2022). In the present cross-sectional model, lower RESE statistically accounted for part of the association between sleep quality problems and lower authentic pride. By contrast, although a small direct association with hubristic pride emerged in the mediation model, the corresponding indirect association through RESE was not significant; therefore, H4b was not supported. The significant indirect association for authentic pride, together with the nonsignificant indirect association for hubristic pride, suggests that an indirect association involving RESE was observed only for authentic pride in the present data. However, because the present analyses did not formally compare the indirect effects for authentic and hubristic pride, this outcome-specific interpretation should be treated cautiously. The findings are consistent with the theoretical view that authentic pride is grounded in competence and self-mastery, whereas hubristic pride is more closely related to superiority-oriented and defensive self-evaluation (Tracy and Robins, 2007).

Tai Chi experience also moderated the indirect association between sleep quality problems and authentic pride through RESE, supporting H5a. Although prior research has linked Tai Chi to psychological wellbeing, stress reduction, sleep quality, and emotion regulation (Kraft et al., 2024; Si et al., 2020; Wang et al., 2010; Yin et al., 2023), the present findings did not support a protective or buffering interpretation. Instead, the negative association between sleep quality problems and RESE was stronger among participants with relatively longer Tai Chi experience and was accompanied by a stronger conditional indirect association with authentic pride. The observed moderation may be understood in relation to the mind–body characteristics of Tai Chi, although this interpretation must remain cautious. Tai Chi practice repeatedly directs attention to breathing, postural control, movement awareness, balance, and internal bodily states. Longer practice experience may therefore represent greater exposure to a context in which bodily conditions are closely connected with perceived self-regulatory functioning. One possibility is that, among individuals with longer practice histories, sleep-related difficulties are more closely associated with how they appraise their capacity to regulate emotions. However, because the present study measured Tai Chi experience only as years of practice and did not directly assess interoceptive awareness, mindful engagement, or practice quality, this interpretation should be regarded as theoretically informed rather than as evidence of a specific mechanism (Lazzarelli et al., 2024; Price and Hooven, 2018; Yeh et al., 2014).

In contrast, Tai Chi experience did not significantly moderate the indirect association between sleep quality problems and hubristic pride through RESE, meaning that H5b was not supported. This result is consistent with the mediation findings. Although Tai Chi experience moderated the association between sleep quality problems and RESE, the indirect association through RESE was not statistically significant for hubristic pride. Accordingly, a conditional indirect association was supported for authentic pride, but not for hubristic pride. However, because the present analyses did not formally compare the moderated mediation indices for the two pride outcomes, this interpretation should be treated cautiously. Conceptually, this pattern remains consistent with the view that authentic pride may be closely tied to competence and self-mastery, whereas hubristic pride may be closely tied to superiority, status orientation, and inflated self-evaluation (Cheng et al., 2010; Tracy and Robins, 2007).

These findings open a more nuanced view of how bodily recovery, self-regulatory beliefs, and self-evaluative emotions are connected. Theoretically, they extend pride research by suggesting that authentic pride may be linked not only to achievement or status-related processes, but also to sleep-related bodily recovery and perceived emotion-regulation capacity. Practically, the findings suggest that sleep quality problems may be relevant in Tai Chi and other mind–body practice contexts because sleep quality problems were associated with lower regulatory emotional self-efficacy, which was also associated with lower authentic pride in the specified cross-sectional model. However, the moderating role of Tai Chi experience should not be interpreted as evidence of a protective training effect. Rather, it suggests that accumulated practice experience may condition how sleep-related difficulties are connected with self-regulatory beliefs.

### Limitations and future research

Several limitations should be acknowledged. First, the cross-sectional design precludes causal inference; the present data cannot determine the temporal ordering among sleep quality problems, RESE, and pride (Maxwell and Cole, 2007). Relatedly, because the PSQI assessed sleep quality problems over the past month whereas the AHPS indexed dispositional pride-proneness, the findings should be interpreted as cross-level associations rather than as evidence concerning momentary pride states. Although a sensitivity check was conducted for the primary regression-path findings, the bootstrapped indirect effects and indices of moderated mediation were interpreted using conventional 95% confidence intervals; therefore, findings close to the conventional *p* < 0.05 threshold should be interpreted cautiously. Second, all core constructs were assessed using self-report measures, which may introduce shared method variance and subjective response biases (Podsakoff et al., 2003), particularly because sleep quality problems and RESE both involve subjective appraisals of bodily and emotional states. Accordingly, although the Harman single-factor analysis indicated that no single factor dominated the covariance structure, the analysis does not exclude the possibility of common method effects. In addition, the internal consistency of the PSQI global score was modest in the present sample. Although the PSQI is commonly used as a global index of subjective sleep quality, previous psychometric evidence suggests that its global score is better interpreted as a multidimensional indicator rather than as a unidimensional reflective scale (Buysse et al., 1989; Zhang et al., 2020). Therefore, associations involving sleep quality problems should be interpreted cautiously as reflecting overall subjective sleep difficulties. Third, Tai Chi experience was operationalized as years of practice, which does not capture current practice frequency, intensity, instructional quality, practice depth, mindful engagement, interoceptive awareness, or body awareness. Thus, the moderating role of Tai Chi experience should be interpreted as a contextual association rather than as evidence of a dose–response training effect or enhanced mind–body awareness. Fourth, the study did not assess participants’ student status, academic major, competitive status, athletic identity, martial arts background, or instructional level, which may be relevant to pride and self-regulatory beliefs. Accordingly, the extent to which the sample comprised university students, student-athletes, or students majoring in sport or martial arts could not be determined. Finally, the sample consisted of relatively young Chinese Tai Chi practitioners with regular and sustained practice, recruited from organized club- and class-based settings, including university-based courses, which may limit the generalizability of the findings to older practitioners, occasional practitioners, clinical populations, or Tai Chi practitioners outside organized club- or class-based settings. Given the mean age and male predominance of the sample, the findings should not be assumed to generalize across different gender distributions, age groups, cultural or geographic backgrounds, or the broader population of community-dwelling Tai Chi practitioners.

Future research should use longitudinal, experimental, or daily-diary designs to clarify the temporal ordering among sleep quality problems, RESE, and pride. Future studies should also include more balanced age groups, especially middle-aged and older practitioners, to determine whether the observed conditional process differs across developmental stages. Future studies should also recruit samples with more balanced gender distributions and from diverse cultural and geographic settings to determine whether the observed associations are consistent across different population backgrounds. Studies could also combine self-report data with objective or repeated sleep indicators, such as actigraphy, sleep diaries, or ecological momentary assessment. In addition, future work should distinguish Tai Chi practice years from current practice frequency, intensity, quality, instructional context, mindful engagement, interoceptive awareness, and body awareness to clarify why the sleep quality problems–RESE association was stronger among more experienced practitioners. Future studies should also examine whether this pattern differs across recreational practitioners, competitive martial arts practitioners, older community practitioners, clinical populations, or non-Tai-Chi populations. Finally, because the indirect association involving RESE was not statistically significant for hubristic pride, future research should examine alternative mechanisms, such as social comparison, superiority beliefs, dominance motives, defensive self-esteem, narcissistic tendencies, or self-enhancement processes.

## Conclusion

The present study found that sleep quality problems were indirectly associated with lower authentic-pride proneness through lower regulatory emotional self-efficacy. The corresponding indirect association was not statistically significant for hubristic pride. The conditional indirect association with authentic pride also varied according to Tai Chi experience, but the observed pattern was not consistent with a protective interpretation. Instead, the negative association between sleep quality problems and regulatory emotional self-efficacy was stronger among participants with longer Tai Chi experience. Given the cross-sectional design and the measurement of Tai Chi experience as years of practice, this finding should be interpreted as a contextual association rather than evidence of a training effect. Overall, in this predominantly young sample of Chinese Tai Chi practitioners with regular practice experience, the study highlights regulatory emotional self-efficacy as a plausible psychological correlate within the association between sleep quality problems and authentic pride proneness.

## Data Availability

The datasets generated and/or analysed during the current study are not publicly available due to ethical and privacy restrictions but are available from the corresponding author on reasonable request.
